# Epidemiology of congenital polydactyly and syndactyly in Hunan Province, China

**DOI:** 10.1186/s12884-024-06417-y

**Published:** 2024-03-23

**Authors:** Xu Zhou, Ting Li, Haiyan Kuang, Ying Zhou, Donghua Xie, Jian He, Juan Xiao, Chanchan Chen, Yurong Jiang, Junqun Fang, Hua Wang

**Affiliations:** 1 Hunan Provincial Maternal and Child Health Care Hospital, Changsha, Hunan Province 410000 China; 2 The Hunan Children’s Hospital, Changsha, Hunan Province 410000 China; 3 National Health Commission Key Laboratory of Birth Defects Research, Prevention and Treatment, Hunan Provincial Maternal and Child Health Care Hospital, Changsha, Hunan 410000 China

**Keywords:** Polydactyly, Syndactyly, Prevalence, Sex, Residence characteristics, Maternal age

## Abstract

**Objective:**

To describe the prevalence and epidemiology of congenital polydactyly and syndactyly in Hunan Province, China, 2016–2020.

**Methods:**

Data were obtained from the Birth Defects Surveillance System in Hunan Province, China, 2016–2020. Prevalence of birth defects (polydactyly or syndactyly) is the number of cases per 1000 births (**unit: ‰**). Prevalence and 95% confidence intervals (CI) were calculated by the log-binomial method. Chi-square trend tests (*χ*^*2*^_*trend*_) were used to determine trends in prevalence by year. Crude odds ratios (ORs) were calculated to examine the association of each demographic characteristic with polydactyly and syndactyly.

**Results:**

Our study included 847,755 births, and 14,459 birth defects were identified, including 1,888 polydactyly and 626 syndactyly cases, accounting for 13.06% and 4.33% of birth defects, respectively. The prevalences of total birth defects, polydactyly, and syndactyly were 17.06‰ (95%CI: 16.78–17.33), 2.23‰ (95%CI: 2.13–2.33), and 0.74‰ (95%CI: 0.68–0.80), respectively. Most polydactyly (96.77%) and syndactyly (95.69%) were diagnosed postnatally (within 7 days). From 2016 to 2020, the prevalences of polydactyly were 1.94‰, 2.07‰, 2.20‰, 2.54‰, and 2.48‰, respectively, showing an upward trend (χ^2^_trend_ = 19.48, *P* < 0.01); The prevalences of syndactyly were 0.62‰, 0.66‰, 0.77‰, 0.81‰, and 0.89‰, respectively, showing an upward trend (χ^2^_trend_ = 10.81, *P* = 0.03). Hand polydactyly (2.26‰ vs. 1.33‰, OR = 1.69, 95%CI: 1.52–1.87) and hand syndactyly (0.43‰ vs. 0.28‰, OR = 1.42, 95%CI: 1.14–1.76) were more common in males than females. Polydactyly (2.67‰ vs. 1.93‰, OR = 1.38, 95%CI: 1.26–1.51) and syndactyly (0.91‰ vs. 0.62‰, OR = 1.47, 95%CI: 1.26–1.72) were more common in urban areas than in rural areas. Compared to maternal age 25–29, hand polydactyly was more common in maternal age < 20 (2.48‰ vs. 1.74‰, OR = 1.43, 95%CI: 1.01–2.02) or ≥ 35 (2.25‰ vs. 1.74‰, OR = 1.30, 95%CI: 1.12–1.50).

**Conclusion:**

In summary, we have described the prevalence and epidemiology of polydactyly and syndactyly from hospital-based surveillance in Hunan Province, China, 2016–2020. Our findings make some original contributions to the field, which may be valuable for future research.

## Introduction

Birth defects are structural or functional anomalies at or before birth [1]. The accepted prevalence of birth defects is about 2–3% worldwide [2]. Polydactyly refers to a birth defect of the hand or foot marked by the presence of supernumerary digits [3]. Syndactyly refers to a birth defect of the hand or foot marked by the webbing between adjacent fingers or toes [4]. The globally accepted prevalences of polydactyly and syndactyly were 0.3–3.6 and 0.3–1 per 1000 births, respectively [5, 6]. Polydactyly and syndactyly are the most common limb-related birth defects [5, 7] and one of the most common birth defects [8, 9]. Polydactyly and syndactyly cause cosmetic and functional impairments and may be associated with some syndromes [10–12], which may be a significant burden on the patients and their families. Therefore, studies on polydactyly and syndactyly are significant and deserve more attention.

There were some studies on the prevalence and epidemiology of polydactyly and syndactyly. E.g., the prevalences of polydactyly and syndactyly in China were 0.945‰ and 0.31‰, respectively [13, 14], in New York State were 2.34‰ and 0.74‰, respectively [15, 16], in South Korea were 1.157‰ and 0.309‰, respectively [17], in southern Thailand were 0.32‰ and 0.21‰, respectively [18]. Xiang et al. found that the prevalence of polydactyly was higher in males than females [19]. Dai et al. found that syndactyly was more common in urban than rural regions [14]. Zhou et al. found that the prevalence of polydactyly increased with maternal age [13]. There are huge variations in the prevalence and epidemiology of these reports, which were thought to be related to genetic mutations [5, 20] or environmental, extragenic, and stochastic factors [19, 21–23]. **However**, there are limitations in many previous studies. **First**, some studies had data limitations, such as relatively few cases included or surveys conducted in unrepresentative districts or hospitals, which may also contribute to the differences in the prevalence and epidemiology in different studies. **Second**, although some studies have reported the overall prevalence of polydactyly or syndactyly, few studied the prevalence and epidemiology of polydactyly or syndactyly in-depth, such as a comprehensive description and comparison of the prevalence of various specific types. **Third**, there are few systematic studies on polydactyly and syndactyly in China. **Fourth**, some studies needed to be updated.

Therefore, we conducted a comprehensive analysis based on hospital-based surveillance in Hunan Province, China, 2016–2020, to describe the prevalence and epidemiology of polydactyly and syndactyly, which may make some original contributions to the field.

## Methods

### Data sources

This study used data from the Birth Defects Surveillance System in Hunan Province, China, 2016–2020, which is run by the Hunan Provincial Health Commission and involves 52 representative registered hospitals in Hunan Province. In 1996, the Hunan Provincial Health Commission selected those hospitals as surveillance sites, which had undergone a comprehensive evaluation process by experts before the decision. Those 52 hospitals are distributed evenly throughout the province’s municipalities and have well-established services for diagnosing and registering birth defects. Live births in those hospitals account for approximately 1/4 of the total live births in the province. The surveillance population included all births (including live births, deaths, and legal termination of pregnancy at 28 weeks of gestation and beyond) and birth defects (between 28 weeks of gestation and seven days after delivery) in the surveillance sites. Surveillance data of births and birth defects included demographic characteristics such as sex, residence, maternal age (age of the mother became pregnant), and other key information.

The Birth Defects Surveillance System diagnosed and classified birth defects according to the International Classification of Disease, Tenth Revision (ICD-10). The ICD code for birth defects is Q00-Q99, polydactyly is Q69, and syndactyly is Q70. Polydactyly or syndactyly will be further classified into hand polydactyly (or syndactyly) and foot polydactyly (or syndactyly) according to where they occurred.

### Definitions

Prevalence of birth defects (polydactyly or syndactyly) is the number of cases per 1000 births (**unit: ‰**). Perinatal deaths include stillbirths (fetal deaths with a gestation of 28 weeks or more) and early neonatal deaths (infant deaths less than 7 days of age). The perinatal mortality rate is the number of perinatal deaths per 100 births.

### Informed consents

We confirmed that informed consent was obtained from all subjects and/or their legal guardian(s). Doctors obtain consent from pregnant women before collecting surveillance data, witnessed by their families and the heads of the obstetrics or neonatal departments. Doctors obtain consent from their parents or guardians for live births, witnessed by their families and the heads of the obstetrics or neonatal departments. Since the Health Commission of Hunan Province collects those data, and the government has emphasized the privacy policy in the “Maternal and Child Health Monitoring Manual in Hunan Province”, there is no additional written informed consent.

### Ethics guideline statement

The Medical Ethics Committee of Hunan Provincial Maternal and Child Health Care Hospital approved the study. (NO: 2022-S65). It is a retrospective study of medical records; all data were fully anonymized before we accessed them. Moreover, we de-identified the patient records before analysis. We confirmed that all methods were performed following the relevant guidelines and regulations.

### Data quality control

The Health Commission of Hunan Province formulated the Work Manual of Hospital Surveillance of Birth Defects in Hunan Province as the work standard for the whole province. Data were collected and reported by experienced doctors. To reduce the integrity and information error rates, we asked the technical guidance departments to carry out comprehensive quality control each year.

### Statistical analysis

Prevalence and 95% confidence intervals (CI) were calculated by the log-binomial method [24]. Chi-square trend tests (*χ*^*2*^_*trend*_) were used to determine trends in prevalence by year. *P* < 0.05 was considered statistically significant. Crude odds ratios (ORs) were calculated to examine the association of each demographic characteristic with polydactyly and syndactyly.

Statistical analyses were performed using SPSS 18.0 (IBM Corp., NY, USA).

## Results

### Prevalence of total birth defects, polydactyly and syndactyly in Hunan Province, China, 2016–2020

Our study included 847,755 births, and 14,459 birth defects were identified, including 1,888 polydactyly and 626 syndactyly cases, accounting for 13.06% and 4.33% of birth defects, respectively. The prevalences of total birth defects, polydactyly, and syndactyly were 17.06‰ (95%CI: 16.78–17.33), 2.23‰ (95%CI: 2.13–2.33), and 0.74‰ (95%CI: 0.68–0.80), respectively. A total of 52 cases were polydactyly of both hand and foot, and 36 were syndactyly of both hand and foot.

From 2016 to 2020, the prevalences of birth defects were 18.20‰, 18.00‰, 16.31‰, 16.03‰, and 16.47‰, respectively, showing a downward trend (χ^2^_trend_ = 30.83, *P* < 0.01); The prevalences of polydactyly were 1.94‰, 2.07‰, 2.20‰, 2.54‰, and 2.48‰, respectively, showing an upward trend (χ^2^_trend_ = 19.48, *P* < 0.01); The prevalences of syndactyly were 0.62‰, 0.66‰, 0.77‰, 0.81‰, and 0.89‰, respectively, showing an upward trend (χ^2^_trend_ = 10.81, *P* = 0.03). (Table 1)

**Table 1:** Prevalence of total birth defects, polydactyly and syndactyly in Hunan Province, China, 2016–2020

Year	Births (n)	Total birth defects		Polydactyly			Syndactyly	
		n	Prevalence (‰,95%CI)	n		Prevalence (‰,95%CI)	n	Prevalence (‰,95%CI)
2016	170,688	3107	18.20(17.56–18.84)	331		1.94(1.73–2.15)	105	0.62(0.50–0.73)
2017	196,316	3533	18.00(17.40-18.59)	406		2.07(1.87–2.27)	129	0.66(0.54–0.77)
2018	177,762	2900	16.31(15.72–16.91)	391		2.20(1.98–2.42)	136	0.77(0.64–0.89)
2019	164,840	2643	16.03(15.42–16.65)	418		2.54(2.29–2.78)	133	0.81(0.67–0.94)
2020	138,149	2276	16.47(15.80-17.15)	342		2.48(2.21–2.74)	123	0.89(0.73–1.05)
Total	847,755	14,459	17.06(16.78–17.33)	1888		2.23(2.13–2.33)	626	0.74(0.68–0.80)

*Abbreviations* CI = confidence interval

The number of hand polydactyly, foot polydactyly, hand syndactyly, and foot syndactyly were 1597, 343, 342, and 320, respectively, and the prevalences were 1.88‰ (95%CI: 1.79–1.98), 0.40‰ (95%CI: 0.36–0.45), 0.40‰ (95%CI: 0.36–0.45), and 0.38‰ (95%CI: 0.34–0.42), respectively. And 5.72% (108 cases) of polydactyly and 5.91% (37 cases) of syndactyly were combined with other defects. (Table 2)

**Table 2:** Prevalence of polydactyly and syndactyly by subtypes

Types	n	Prevalence (‰,95%CI)
Polydactyly	1888	2.23(2.13–2.33)
Hand polydactyly	1597	1.88(1.79–1.98)
Foot polydactyly	343	0.40(0.36–0.45)
Syndactyly	626	0.74(0.68–0.80)
Hand syndactyly	342	0.40(0.36–0.45)
Foot syndactyly	320	0.38(0.34–0.42)

*Note* 52 cases were polydactyly of both hand and foot, and 36 cases were syndactyly of both hand and foot*Abbreviations* CI = confidence interval

### Prevalence of polydactyly and syndactyly by sex

Both polydactyly (2.71‰ vs. 1.69‰, OR = 1.60, 95%CI: 1.46–1.76) and syndactyly (0.84‰ vs. 0.62‰, OR = 1.35, 95%CI:1.15–1.58) were more common in males than females. Both hand polydactyly (2.26‰ vs. 1.33‰, OR = 1.69, 95%CI: 1.52–1.87) and hand syndactyly (0.43‰ vs. 0.28‰, OR = 1.42, 95%CI: 1.14–1.76) were more common in males than females, while no significant differences in the prevalence of foot polydactyly (0.44‰ vs. 0.36‰) or foot syndactyly (0.41‰ vs. 0.34‰) between males than females (The 95%CI for OR contains 1). (Table 3)

**Table 3:** Prevalence of polydactyly and syndactyly by sex

Types	Male (N: 448,288)		Female (N: 399,368)		OR(95%CI) (Reference: females)
	n	Prevalence (‰,95%CI)	n	Prevalence (‰,95%CI)	
Polydactyly	1213	2.71(2.55–2.86)	674	1.69(1.56–1.82)	1.60(1.46–1.76)
Hand polydactyly	1044	2.33(2.19–2.47)	552	1.38(1.27–1.50)	1.69(1.52–1.87)
Foot polydactyly	198	0.44(0.38–0.50)	144	0.36(0.30–0.42)	1.23(0.99–1.52)
Syndactyly	377	0.84(0.76–0.93)	249	0.62(0.55–0.70)	1.35(1.15–1.58)
Hand syndactyly	210	0.47(0.41–0.53)	132	0.33(0.27–0.39)	1.42(1.14–1.76)
Foot syndactyly	183	0.41(0.35–0.47)	137	0.34(0.29–0.40)	1.19(0.95–1.49)

*Abbreviations* N = number of births; CI = confidence interval; OR = odds ratio

### Prevalence of polydactyly and syndactyly by residence

Both polydactyly (2.67‰ vs. 1.93‰, OR = 1.38, 95%CI: 1.26–1.51) and syndactyly (0.91‰ vs. 0.62‰, OR = 1.47, 95%CI: 1.26–1.72) were more common in urban areas than in rural areas. When categorized by hand and foot, polydactyly or syndactyly was also more common in urban than rural areas in all groups (OR > 1, *P* < 0.05). (Table 4)

**Table 4:** Prevalence of polydactyly and syndactyly by residence

Types	Urban (N: 342,178)		Rural (N: 505,577)		OR(95%CI) (Reference: rural)
	n	Prevalence (‰,95%CI)	n	Prevalence (‰,95%CI)	
Polydactyly	912	2.67(2.49–2.84)	976	1.93(1.81–2.05)	1.38(1.26–1.51)
Hand polydactyly	776	2.27(2.11–2.43)	821	1.62(1.51–1.73)	1.40(1.27–1.54)
Foot polydactyly	158	0.46(0.39–0.53)	185	0.37(0.31–0.42)	1.26(1.02–1.56)
Syndactyly	312	0.91(0.81–1.01)	314	0.62(0.55–0.69)	1.47(1.26–1.72)
Hand syndactyly	171	0.50(0.42–0.57)	171	0.34(0.29–0.39)	1.48(1.20–1.83)
Foot syndactyly	154	0.45(0.38–0.52)	166	0.33(0.28–0.38)	1.37(1.10–1.71)

*Abbreviations* N = number of births; CI = confidence interval; OR = odds ratio

### Prevalence of polydactyly and syndactyly by maternal age

For maternal age < 20, 20–24, 25–29, 30–34, and ≥ 35, the prevalences of polydactyly were 2.77‰, 2.11‰, 2.08‰, 2.28‰, 2.64‰, respectively, and the prevalences of syndactyly were 0.58‰, 0.82‰, 0.68‰, 0.76‰ and 0.81‰, respectively. Compared to maternal age 25–29, polydactyly was more common in maternal age ≥ 35 (2.64‰ vs. 2.08‰, OR = 1.27, 95%CI: 1.11–1.45), and hand polydactyly was more common in maternal age < 20 (2.48‰ vs. 1.74‰, OR = 1.43, 95%CI: 1.01–2.02) or ≥ 35 (2.25‰ vs. 1.74‰, OR = 1.30, 95%CI: 1.12–1.50). There were no significant differences in the prevalence of syndactyly or foot polydactyly among different maternal age groups (Reference: maternal age 25–29) (The 95%CI for OR contains 1). (Table 5)

**Table 5:** Prevalence of polydactyly and syndactyly by maternal age

Types	< 20 years old (N: 13,711)			20–24 years old (N: 118,531)			25–29 years old (N: 357,582) (Reference)		30–34 years old (N: 243,649)			≥ 35 years old (N: 114,282)		
	n	Prevalence (‰,95%CI)	OR (95%CI)	n	Prevalence (‰,95%CI)	OR (95%CI)	n	Prevalence (‰,95%CI)	n	Prevalence (‰,95%CI)	OR (95%CI)	n	Prevalence (‰,95%CI)	OR (95%CI)
Polydactyly	38	2.77(1.89–3.65)	1.33(0.96–1.85)	250	2.11(1.85–2.37)	1.02(0.88–1.17)	743	2.08(1.93–2.23)	555	2.28(2.09–2.47)	1.10(0.98–1.22)	302	2.64(2.34–2.94)	1.27(1.11–1.45)
Hand polydactyly	34	2.48(1.65–3.31)	1.43(1.01–2.02)	206	1.74(1.50–1.98)	1.00(0.85–1.17)	621	1.74(1.60–1.87)	479	1.97(1.79–2.14)	1.13(1.00-1.28)	257	2.25(1.97–2.52)	1.30(1.12–1.50)
Foot polydactyly	4	0.29(0.01–0.58)	0.75(0.28–2.01)	48	0.40(0.29–0.52)	1.03(0.75–1.44)	140	0.39(0.33–0.46)	93	0.38(0.30–0.46)	0.97(0.75–1.27)	58	0.51(0.38–0.64)	1.30(0.95–1.76)
Syndactyly	8	0.58(0.18–0.99)	0.86(0.42–1.74)	97	0.82(0.66–0.98)	1.20(0.95–1.52)	243	0.68(0.59–0.77)	185	0.76(0.65–0.87)	1.12(0.92–1.35)	93	0.81(0.65–0.98)	1.20(0.94–1.52)
Hand syndactyly	1	0.07(-0.07-0.22)	0.20(0.03–1.40)	51	0.43(0.31–0.55)	1.16(0.84–1.60)	133	0.37(0.31–0.44)	103	0.42(0.34–0.50)	1.14(0.88–1.47)	54	0.47(0.35–0.60)	1.27(0.93–1.74)
Foot syndactyly	7	0.51(0.13–0.89)	1.47(0.69–3.15)	48	0.40(0.29–0.52)	1.17(0.84–1.63)	124	0.35(0.29–0.41)	91	0.37(0.30–0.45)	1.08(0.82–1.41)	50	0.44(0.32–0.56)	1.26(0.91–1.75)

*Note* maternal age 25–29 is the **reference** for calculating the OR values *Abbreviation*SN = number of births; CI = confidence interval; OR = odds ratio

### Perinatal deaths and time of diagnosis for polydactyly and syndactyly

A total of 29 perinatal deaths attributable to polydactyly were identified, including 28 stillbirths and 1 early neonatal death (attributable to hand polydactyly), and 20 stillbirths were selective termination of pregnancy. A total of 23 perinatal deaths attributable to syndactyly were identified, and all of them were stillbirths, and 17 stillbirths were selective termination of pregnancy. The perinatal mortality rates of polydactyly and syndactyly were 1.54% and 3.67%, respectively, with significant differences in the prevalence (*χ*^*2*^ = 10.61, *P* = 0.001). Table 6 shows the details of perinatal deaths from polydactyly and syndactyly. (Table 6)

**Table 6:** Perinatal deaths from polydactyly and syndactyly

Types	n	Perinatal deaths (n)	Perinatal mortality rate (%)
Polydactyly	1888	29	1.54
Hand polydactyly	1597	23	1.44
Foot polydactyly	343	10	2.92
Syndactyly	626	23	3.67
Hand syndactyly	342	19	5.56
Foot syndactyly	320	9	2.81

Most polydactyly (96.77%) and syndactyly (95.69%) were diagnosed postnatally (within 7 days). Table 7 shows the details of the time of diagnosis for polydactyly and syndactyly. (Table 7)

**Table 7:** Time of diagnosis for polydactyly and syndactyly

Types	n	Prenatal diagnosis (n)	Proportion (%)	Postnatal diagnosis (within 7 days)	Proportion (%)
Polydactyly	1888	61	3.23	1827	96.77
Hand polydactyly	1597	48	3.01	1549	96.99
Foot polydactyly	343	21	6.12	322	93.88
Syndactyly	626	27	4.31	599	95.69
Hand syndactyly	342	20	5.85	322	94.15
Foot syndactyly	320	12	3.75	308	96.25

## Discussion

Overall, we have described the prevalence and epidemiology of polydactyly and syndactyly. Our study is the most recent comprehensive study on the prevalence and epidemiology of polydactyly and syndactyly from long-term hospital-based surveillance data, which makes some original contributions to the field.

There were several meaningful findings. **First**, in this study, the prevalences of polydactyly and syndactyly were 2.23‰ and 0.74‰, respectively, which was within the globally acceptable range (The globally accepted prevalences of polydactyly and syndactyly were 0.3–3.6 and 0.3–1 per 1000 births, respectively [5, 6]). **However**, there were huge variations in the reported prevalences of polydactyly and syndactyly in different countries. In contrast, the variations between different regions in China were relatively small, as shown in Table 8 [8, 13–18, 25–29]. We believed these differences were mainly related to ethnicity and genetics [5, 30, 31]. **In addition**, data sources may also contribute to the differences, as many studies were based on relatively few cases included or surveys conducted in unrepresentative districts or hospitals.

**Table 8:** Prevalence of polydactyly and syndactyly in different countries and regions

Country	Regions	Title	Data source	Year	Polydactyly prevalence	Syndactyly prevalence
United States	New York State	The Prevalence of Congenital Hand and Upper Extremity Anomalies Based Upon the New York Congenital Malformations Registry	New York Congenital Malformations Registry database	1992–2010	2.34‰	0.13‰
United States	New York State	Epidemiology of syndactyly in New York State	New York State Statewide Planning and Research Cooperative System	1997–2014		0.74‰
Europe		Trends in congenital anomalies in Europe from 1980 to 2012	61 congenital anomaly subgroups (excluding chromosomal) in 25 population-based EUROCAT registries	1980–2012		0.486‰
Korea		Epidemiology of congenital upper limb anomalies in Korea: A nationwide population-based study	Health Insurance Review and Assessment Service of Korea	2007–2016	1.157‰	0.309‰
Israel		Polydactyly in the multiethnic ‘Negev’ population at southern Israel	A retrospective analysis of 189 polydactyly patients	2014	0.5‰	
Thailand	In 3 provinces	Prevalence of congenital limb defects: Data from birth defects registries in three provinces in Southern Thailand	Population-based birth defects registries	2009–2013	0.32‰	0.21‰
Argentina	Buenos Aires	Birth prevalence of congenital anomalies in the City of Buenos Aires, Argentina, according to socioeconomic level	In hospitals of the City of Buenos Aires	2010–2016	0.69‰	
China		Epidemiological analysis of polydactylies in Chinese perinatals	Hospital-based surveillance within Chinese Birth Defects Monitoring Network	1996–2000	0.945‰	
China		Epidemiological analysis of syndactyly in Chinese perinatals	Hospital-based surveillance within Chinese Birth Defects Monitoring Network	1987–2001		0.31‰
China	Tongzhou District in Beijing City	Prevalence of birth defects in the Tongzhou District of Beijing between 2006 and 2012	Hospital-based birth defects surveillance	2006–2012	1.73‰	0.73‰
China	Guilin	Birth defects data from hospital-based birth defect surveillance in Guilin, China, 2018–2020	Hospital-based birth defects surveillance	2018–2020	3.24‰	1.14‰
China	In a District of Southern Jiangsu	Birth Defects Data From Population-Based Birth Defects Surveillance System in a District of Southern Jiangsu, China, 2014–2018	Population-Based Birth Defects Surveillance	2014–2018	1.961‰	0.642‰

**Second**, from 2016 to 2020, the prevalence of birth defects showed a downward trend, while the prevalences of polydactyly and syndactyly showed upward trends. The downward trend in the prevalence of birth defects may be mainly related to improvements in prenatal screening and diagnosis technologies, causing more and more birth defects diagnosed early in pregnancy (before 28 weeks of gestation) and selective termination, which were not used to calculate the prevalence of birth defects. E.g., most Down syndromes are diagnosed and terminated in the second trimester due to prenatal screening and diagnosis [32]. The prevalence of Down syndrome was 1.49 per 10,000 fetuses in Hunan Province, China, 2010–2020 [33], which was significantly lower than the accepted prevalence (almost 1 in 600 live births) [34]. **In comparison**, most polydactyly and syndactyly were diagnosed postnatally, and few perinatal deaths were associated with polydactyly and syndactyly. **Moreover**, we infer that the upward trends in the prevalences of polydactyly and syndactyly may be related to some other factors, such as China’s two-child policy since 2014 [35], number of pregnancies, socioeconomic conditions, et al., which were rarely addressed in previous studies. Our findings provide clues for future research.

**Third**, polydactyly and syndactyly were more common in males than females, consistent with most previous studies in China [13, 14, 19, 36, 37] and also some other countries, such as South Korea [17] and Ireland [38]. **However**, polydactyly and syndactyly were more common in females than males in some Middle Eastern and European countries [39–41]. **In addition**, hand polydactyly and hand syndactyly were more common in males than females. However, there were no significant differences in the prevalence of foot polydactyly or foot syndactyly between males and females. It indicates that the higher prevalence of polydactyly (or syndactyly) in males may be caused mainly by hand polydactyly (or syndactyly) but not foot polydactyly (or syndactyly). **Overall**, the mechanisms of this phenomenon are unclear. As discussed above, these differences may be mainly related to differences in ethnicity and genetics.

**Fourth**, polydactyly and syndactyly were more common in urban areas than rural areas. There were also different results from different studies. E.g., Dai et al. found a higher prevalence of syndactyly in urban areas [14]; Zhou et al. found no significant difference in the prevalence of polydactyly between urban and rural areas [13]. There are several reasons for this phenomenon. **On the one hand**, due to differences in socioeconomic conditions between urban and rural areas, there may be differences in hospital delivery rates and diagnosis rates [42]. It is also the reason for many specific defects, such as congenital heart defects, hypospadias, cleft palate, and Down syndrome, which are more common in urban areas than in rural areas [33, 43]. **On the other hand**, differences in some factors between urban and rural areas may also contribute to polydactyly and syndactyly, such as air pollution and hazardous chemicals [22, 44, 45]. **However**, those factors were not included in our study due to data limitations, which were rarely addressed in previous studies. Our findings provide clues for future research.

**Fifth**, low (< 20) or advanced (≥ 35) maternal age were associated with polydactyly. Several studies also found higher prevalences of polydactyly in low maternal age [33, 46, 47]. Jennita et al. found that low maternal age was not associated with polydactyly after adjusting for parity [47]. **However**, few studies reported higher prevalences of polydactyly in advanced maternal age. **In addition**, the occurrence of syndactyly appeared independent of maternal age, consistent with several previous studies [33, 48]. **However**, Hay et al. found a positive relation between increasing maternal age and increasing prevalence of syndactyly [49]. It indicates that low or advanced maternal age may contribute to those results, or some risk factors are more common in low or advanced maternal age, while maternal age is a confounding factor. **Moreover**, the higher prevalence of polydactyly in low or advanced maternal age may be caused mainly by hand polydactyly but not foot polydactyly, which was similar to the difference between males and females. Castilla et al. believed that the rudimentary structure of upper limb digits in humans gives less margin for developmental errors and a more common under-ascertainment of defective toes [50]. Our findings seem to support this view. Our findings make some original contributions to the field.

**Some things could be improved in our study. First**, we have realized that a regression analysis of risk factors for congenital malformations (polydactyly and syndactyly) such as male gender, city, and maternal age was important. **However**, since in the Birth Defects Surveillance System, reports of the number of births (mainly grouped by sex, residence, and maternal age) and case cards of congenital malformations were collected separately, we were unable to combine them. **Therefore**, we were unable to perform a regression analysis of risk factors for congenital malformations. Moreover, we were unable to calculate the prevalence of polydactyly and syndactyly by demographic characteristics except for sex, residence, and maternal age. **Second**, some potential factors for polydactyly and syndactyly were not included due to data limitations, such as parity and paternal age. **Third**, many cases had multiple specific defects. However, we did not analyze it. **Fourth**, our study did not provide genetic types for polydactyly and syndactyly. More studies need to be done in the future.

## Conclusion

In summary, we have described the prevalence and epidemiology of polydactyly and syndactyly from hospital-based surveillance in Hunan Province, China, 2016–2020. Our findings make some original contributions to the field, which may be valuable for future research.

## Data Availability

All data generated or analyzed during this study are included in this published article.
